# The Difference in the Bacterial Attachment among *Pratylenchus neglectus* Populations and Its Effect on the Nematode Infection

**DOI:** 10.3390/microorganisms10081524

**Published:** 2022-07-27

**Authors:** Rasha Haj Nuaima

**Affiliations:** Institute for Epidemiology and Pathogen Diagnostics, Julius Kühn Institut, Federal Research Centre for Cultivated Plants, Messeweg 11-12, 38104 Braunschweig, Germany; rasha.haj-nuaima@julius-kuehn.de

**Keywords:** bacterial attachment, *far-1* (fatty acid- and retinol-binding gene), nematode cuticle, populations, *Pratylenchus neglectus*, *Rothia* sp.

## Abstract

Different bacterial isolates attach to the cuticle of plant-parasitic nematodes, affecting their interactions with the host plant. Nematode populations differ in their genetic and cuticle structures, causing variable interactions with host plants and natural enemies. In the current study, attachment assays were carried out to compare the attachment of soil bacteria in general and the bacterial isolate of *Rothia* sp. in particular among geographically diverse populations of *Pratylenchus neglectus*. Biological and molecular assays were further conducted to examine the effect of *Rothia* attachment on nematode penetration into barley roots and to sequence the fatty acid- and retinol-binding gene (*Pn-far-1*). The results showed that nematode populations of *P. neglectus* differed in their bacterial attachment. Soil bacteria and *Rothia* sp. attached specifically to the cuticle of *P. neglectus* and did so differently among the nematode populations. *Rothia* attachment caused a reduction in the infectivity of three nematode populations in barley roots. The sequencing of the *far-1* gene revealed genetic variability within and among *P. neglectus* populations. In conclusion, the interaction between *P. neglectus* and their bacterial attachers occurs in a population-specific manner, elucidating an essential aspect of using biological agents to manage plant-parasitic nematodes. Key Message: 1. Geographically diverse populations of the root lesion nematode *Pratylenchus neglectus* differed in the soil bacterial communities attached to their cuticles. 2. The bacterial isolate of *Rothia* sp. attached to the cuticle of *P. neglectus* and reduced its penetration into the host plant in a population-specific manner. 3. The fatty acid- and retinol-binding gene (*far-1*) varied within and among *P. neglectus* populations with their different bacterial attachment.

## 1. Introduction

Recently, many studies have been conducted to investigate the microbial association to the nematode cuticle and its role in the interplay between nematodes and their host plants [1,2,3].

In this context, different bacterial species were isolated from the cuticles of plant-parasitic nematodes (PPN). For example, the opportunistic parasitic bacterial isolates, *Brevibacillus laterosporus* strain G4 and *Bacillus* sp. B16, target nematodes as one possible nutrient resource, but they can penetrate the cuticle to infect and kill the nematode [4]. The obligate parasites in the genus *Pasteuria* have shown great potential as biocontrol agents toward PPN [5], and the first step in the infection process by *Pasteuria* is the attachment of spores to the nematode cuticle [6].

Some of the attaching bacteria suppress PPN by increasing juvenile mortality (*Microbacterium* spp.), decreasing juvenile motility (*Brevundimonas* sp.), or reducing the egg hatch (*Acinetobacter* sp.) [3]. Interestingly, the bacteria attached to the nematode cuticle can also induce plant defenses against nematode infestation. One example is *Bacillus subtilis*, which induces the systemic resistance of tomato plants against *Meloidogyne incognita* [7]. Other bacterial species, such as *Rothia amarae,* were determined to be the most abundant species attached to the cuticles of juveniles *M. hapla* in suppressive soil [1]. However, the role of *Rothia* spp. in suppressing PPN has not yet been studied.

In an artificial system where the plant is absent, the components of both sides of the nematode–microbe interaction interfere in the attachment process. It has been suggested that chemicals on the spore surface, such as carbohydrates, are involved in the attachment of *Pasteuria penetrans* to the cuticle of PPN [8]. Bacilli pathogenic to PPN, such as *Bacillus anthracis*, intimately contact the nematode cuticle with a hair-like nap on the exosporium of its endosporium. This hair-like nap is composed of a collagen-like glycoprotein called BclA (for Bacillus collagen-like protein of anthracis) [8]. The components on the nematode coat, such as carbohydrate residues, carbohydrate-recognition domains, the 250-kDa antigen, and collagen-like proteins, are involved in the attachment of *P. penetrans* spores to the surface of *Meloidogyne* spp. [8,9]. The diversity in the components included in the nematode–microbe interaction reflects its complexity. It appears that each microbial attacher possesses a specific component that interacts with a specific receptor on the nematode surface. This specificity of the nematode–microbe interaction even appears for populations within each of the nematode or microbe species [10].

The heterogeneity of bacterial surface components was determined within and among different populations of *P. penetrans* [6,11,12]. On the surface of PPN, the carbohydrate moieties displayed variations among populations within a nematode species [13,14]. In addition, the highly diversified collagen families cause variations in the physical and structural characteristics of the cuticle, even among the growth stages of a particular nematode species [15,16]. The ability of the attached microbes to suppress PPN might subsequently vary among individual nematodes and populations of the same species.

Among the most damaging species of PPN is the migratory endoparasite *Pratylenchus neglectus* (Rensch, 1924) Filipjev & Schuurmans Stekhoven, 1941. *P. neglectus* is distributed worldwide and causes yield losses in the production of several important crops, such as cereals [17,18,19]. The life cycle of *Pratylenchus* species, including *P. neglectus,* takes about three to nine weeks, starting with the egg stages and developing into four juvenile stages (J1 to J4) and then adults. *P. neglectus* is considered a monosexual species and reproduces by mitotic parthenogenesis [20]. The eggs are laid inside the root tissues of host plants or in the soil [17].

Populations of *P. neglectus* from different geographical regions display differential host pathogenicity [21]. The variability in the interaction between nematode populations and their host plants is related to the variation in the effector genes among these populations [22].

Because of its role in the development and infection processes of PPN [23], the gene effector of fatty acid- and retinol-binding protein (FAR-1) might mediate the nematode adaptation to the host plant. The variability of the *far-1* gene is consequently highly expected among nematode populations originating from fields different in their crop history and agricultural applications. The *far-1* gene is also involved in the process of bacterial attachment to the cuticle of PPN [24]. It stated that the knockdown of *far-1* caused a reduction in the attachment of *P. penetrans* to *M. incognita*. The putative intraspecific variation in *far-1* segments might subsequently lead to a variable interaction between nematode populations and their antagonists.

Based on this background, the objectives of the present study were addressed to 1., investigate if the attachment of soil bacteria in general and *Rothia* sp., in particular, is different among four geographically diverse populations of *P. neglectus*, 2., investigate if the attachment by *Rothia* sp. affects the nematode infection into the host plant, and 3., investigate if the nematode populations with their different bacterial attachment are genetically distinct.

## 2. Materials and Methods

### 2.1. Nematode Culture

Carrot nematode cultures for four populations of *P. neglectus* were supplied by the German institutions of Julius Kühn Institut in Münster and Braunschweig. The studied nematode populations originate from two locations in Germany (Groß Lüsewitz, Niederhummel), one location in France (Lachadella), and one location in the United Kingdom (Mere). Using the approach described by Boisseau and Sarah 2008 [25], the nematodes, consisting of mixed life stages, were extracted from the old culture. To newly culture the studied populations, the extracted nematodes were surface sterilized, placed on new carrot discs, and incubated at 25 °C for three months. The nematode-surface sterilization was conducted by following the protocol described by Hay 1994 [26] with modification. In brief, nematodes were soaked in Chlorhexidine solution (0.005%) for 15 min, rinsed with sterilized water on a 5 µm sieve, and placed in an antibiotic solution containing Penicillin-G (0.1% *w*/*v*) and Streptomycin sulfate BP (0.1% *w*/*v*) for 16 h and finally washed with sterilized water for three times on a 5 µm sieve.

### 2.2. Testing the Soil Bacterial Attachment to P. neglectus Populations

The freshly hatched nematodes were extracted from the new carrot cultures, surface-sterilized as mentioned above, and stored at 4 °C. To remove the microbial contaminants remaining on the nematode cuticle, two days before the test the nematodes were treated with Chlorhexidine solution (0.005%) for 15 min and washed with sterilized water on a 5 µm mesh three times.

A clayey soil sample of 500 g was amassed from individual cores of a research field at Julius Kühn Institut in Braunschweig. For acquiring a soil solution, 10 g of 0.5 mm sieved soil was mixed with 40 mL sterilized water using a Stomacher blender at 230 rpm for 2 min. The obtained mixture was centrifuged at 500× *g* for 10 min to settle down the soil particles and the individual nematodes that might be existed in the soil. The supernatant was sieved through 5 µm mesh pores to dispose of the remained nematodes in the soil solution. The OD_600_ value of the prepared microbial supernatant was 0.2.

The nematode material of each population used for the study was a mixture of the three juvenile stages (J2, J3, J4) and adults. The attachment test was conducted by baiting 600 sterilized nematodes per population in 600 µL of the prepared microbial supernatant at 28 °C and 150 rpm overnight. The baited nematodes of each population were recovered in the 5 µm sieve and washed with sterilized water three times. On R2A plates (Merck, Germany), 20 replicates of ten nematode individuals from each population were plated and incubated at 28 °C overnight. The colony forming units (CFU) were counted and the mean numbers per population were calculated and reported.

### 2.3. DGGE Fingerprinting of Soil Bacteria Attached to P. neglectus Populations

The DGGE fingerprinting of 16S rRNA was applied to resolve the profiles of soil bacterial communities attached to the nematode cuticles of the studied populations. The baited nematodes were recovered from the soil microbial solution and washed with sterilized water. Then 100 individuals with four replicates for each population were transferred with sterilized water into 1.5 mL tubes to extract the genomic DNA of the attached bacteria. Using the FastDNA SPIN Kit for Soil (MP Biomedicals, Santa Ana, CA, USA) the total microbial DNA was extracted from the microbes-attached nematodes. The approach of PCR-DGGE fingerprinting of 16S rRNA for the attached bacteria was described by Heuer et al., 2001 [27].

### 2.4. Testing the Bacterial Attachment of Rothia sp. to P. neglectus Population

Testing the attachment of *Rothia* sp. (Gram-positive bacterium from Micrococcacea) to the Lachadella population was to examine the specificity of the bacterial attachment of the *P. neglectus*. *Rothia* strain SK5-2.1 with GenBank accession: MN421054.19 extracted from *M. hapla* [3] was used in this test. The attachment of *E. coli* to *P. neglectus* was a control treatment because of the low-level attachment of *E. coli* to the cuticle of PPN [2].

To prepare the bacterial solution, scraped pieces of the permanent culture, stored at −80 °C, were added into a falcon tube containing 4 mL liquid LB broth (Thermo Fisher Scientific, Waltham, MA, USA).

After the overnight incubation at 28 °C, bacterial suspensions for both isolates were centrifuged at 275× *g* for 15 min, followed by discarding the supernatant of the LB and suspending pellets with sterilized water.

The 250 surface-sterilized nematodes were baited overnight into 1.5 mL tubes containing 250 μL of the bacterial solutions OD_600_ = 0.12 (equivalent to 9.216 × 10^8^ and 66 × 10^8^ CFU/mL, respectively, for *Rothia* sp. and *E. coli*) with eight replicates for each isolate.

Following three washes with sterilized water on a 5 μm sieve the treated nematodes were transferred to a new 1.5 tube. For each sample, ten individuals were plated on R2A media (Merck, Darmstadt, Germany) and incubated at 28 °C for two days. The CFU formed by living bacteria attached to the nematode cuticle were evaluated for each treatment. The test was carried out two times separately.

### 2.5. Comparing the Bacterial Attachment of Rothia sp. among P. neglectus Populations

To investigate if the bacterial isolate of *Rothia* sp. attached differently to geographically diverse populations of *P. neglectus*, an attachment test of this bacterium to four ones was conducted by following the procedure mentioned above. This assay was carried out two times separately.

### 2.6. Bioassay to Evaluate the Penetration of P. neglectus Populations into Barley Roots with and without Rothia Attachment

Seeds of barley *Hordeum vulgare* L., cv.Monroe were sterilized by soaking in a detergent of sodium hypochlorite 2% for five minutes and then washed with sterilized water three times. The sterilized seeds were placed in a petri dish containing a half-strength MS medium (Merck, Darmstadt, Germany) for three days. The barley seedling was transferred into a standard petri dish (60 mm × 15 mm, Roth, Graz, Austria) containing 15 mL of 23% Pluronic F127 gel (Sigma-Aldrich, St. Louis, MO, USA). Following the protocol described by Williamson and Čepulytė 2017 [28], the Pluronic F127 gel was prepared to test the nematode penetration into barley roots.

For each population, 1600 nematode individuals were baited into a glass tube containing either 1600 μL of *Rothia* suspension with OD_600_ = 0.12 or 1600 μL sterilized water at 28 °C overnight.

Nematodes baited in *Rothia* suspension were washed three times with sterilized water on a 5 µm sieve and transferred by water to a new glass tube.

For each sample, 200 nematode individuals baited in sterilized water or 200 *Rothia*-attached nematodes from each population were added to the barley seedling and incubated at 22 °C and 60% relative humidity. Each treatment within each population had eight replicated samples. Seven days after the nematode inoculation, the barley roots were harvested and stained in acid fuchsin solution as described by Nuaima et al., 2019 [29]. This bioassay was performed two times independently.

### 2.7. Amplifying far-1 Gene of P. neglectus for Sequence Determination

To provide insight into the genetic basis of differences in the bacterial attachment among nematode populations of *P. neglectus*, the fatty acid- and retinol-binding gene (*far-1*) was PCR-amplified from the genomic DNA of four populations and cloned for sequencing.

For gene sequencing search, the published *far-1* cDNA clone of *Pratylenchus penetrans* (GenBank accession no. KY312539) was the BLAST query to the genome sequences of *P. neglctus* published in the NCBI *Sequence Read Archive* (GenBank accession no. SRX1527786). The sequenced regions from SRX1527786 carrying the serial numbers (87752.1 and 87752.2) were used to design the primer pair Pn.far1f (ATG AGT TGA CCG AGG AGG ACA), Pn.far1r (CCT CCA GGT TCG GCT TCT C).

For each population, the nematode DNA was extracted from 200 sterilized individuals with three replicated samples following the protocol published by Nuaima et al., 2018 [22].

To amplify the *far-1* fragments with an expected product size of 368 bp, the PCR reaction of 25 μL was performed by adding 3 μL of target DNA, 5 μL of 5× GoTaq buffer, 2.5 mM of MgCl_2_, 0.2 mM deoxynucleoside triphosphates, 0.24 mM each of forward and reverse primer and 1 U GoTaq Flexi polymerase (Promega). Cycling conditions for the PCR reaction were as follows: 94 °C for 5 min followed by 40 cycles at 94 °C for 30 s, 57 °C for 30 s and 72 °C or 2 min, with a final extension at 72 °C for 7 min, and cooling to 4 °C. 5 μL of the PCR was loaded on a 3% (*w*/*v*) high-resolution agarose gel and electrophoresed at 80 V for 90 min. The agarose gel stained in 0.5 μg/mL of ethidium bromide was visualized under an ultraviolet transilluminator. To sequence the *far-1* gene, the PCR-amplified fragments had to be cloned. The vector pGEM-T and *Escherichia coli* JM109 high-efficiency competent cells (Promega, Madison, WI, USA) were used to clone the gene fragments amplified from the four populations. Extraction of the plasmids carrying the *far-1* gene was by the Gene JET Plasmid Miniprep kit (Thermo Fisher Scientific, Waltham, MA, USA), followed by the gene sequencing using the vector primers T7 or SP6. A multiple sequence alignment of the sequenced *far-1* segments was created by the Neighbor-Joining tree with 1000 bootstraps, using the CLC Main Workbench 8.1.

### 2.8. Data Analysis

Analyzing the data describing the formed CFU of the attached bacteria and the numbers of nematodes that penetrated barley roots was by using the R software (Dunn’s test after a significant Kruskal-Wallis test).

## 3. Results

### 3.1. The Attachment of Soil Bacteria to Nematode Cuticle of P. neglectus Populations

**Figure 1:**
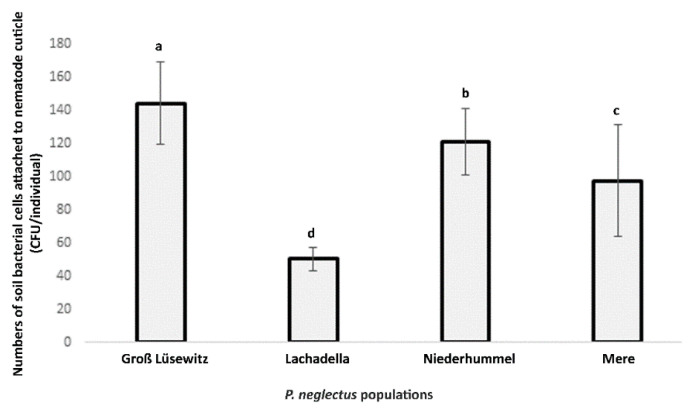
The Bacterial attachment to nematode individuals originated from four populations of *P. neglectus* (Groß Lüsewitz and Niederhummel from Germany, Lachadella from France, and Mere from the United Kingdom). The nematodes were surface sterilized and incubated overnight into microbial suspension extracted from a field soil (OD_600_ = 0.2). Estimating the number of viable microbes attached to nematodes was conducted by counting the CFU formed by plating ten nematodes with 20 replicates on the R2A medium for each population. Different letters indicate significant differences in the counts of CFU/individual among populations (Dunn’s test, *p* < 0.05, *n* = 20).

The attachment assay showed that the numbers of viable bacterial cells attached to the nematode cuticle differed among four geographic populations of *P. neglectus*. The average number of CFU counts per individual was the highest in the Groß Lüsewitz population and lowest in the Lachadella population (Figure 1). The CFU numbers were also variable among individuals within the population. For Groß Lüsewitz, Niederhummel, Lachadella and Mere, the range of CFU values was 18–350, 27–328, 8–135, and 6–654, respectively.

### 3.2. DGGE Fingerprints of Soil Bacteria Attached to P. neglectus Populations

**Figure 2:**
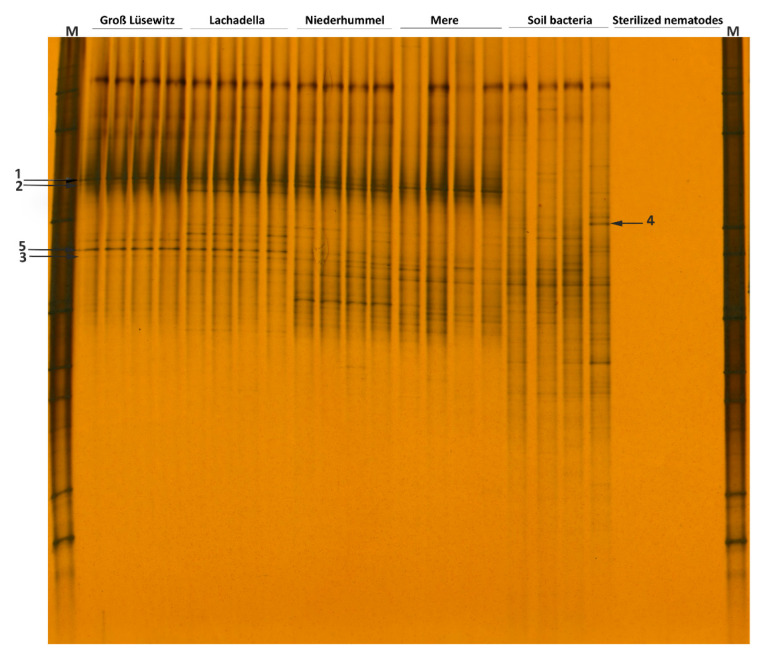
DGGE fingerprints of 16S rRNA gene amplified from the DNA of attached soil bacteria to 100 nematodes per replicate sample of four populations of *P. neglects* (Permutation test on Pearson correlation, *d* = 51, *p* < 0.03). Each electrophoresis band represents a bacterial isolate. The arrows (1, 2, 3) refer to the bacterial isolates commonly amplified from the DNA extracted from the bacteria attached to the nematode cuticle. Arrow (4) refers to the bacterial isolate amplified from the DNA of the soil solution but not from the DNA of attached bacteria. The arrow (5) refers correspondingly to the bacterial isolate amplified with low and high density from the DNA of the soil solution and the attached bacteria.

The DGGE fingerprints of 16S rRNA for the soil bacteria attached to 100 nematode individuals per replicate sample were distinct among the four populations. The populations have only three associated bacterial isolates in common, and each had a unique 16S rRNA profile for the attached bacteria (Figure 2).

The DGGE profiles for the attached bacteria to the four nematode populations are distinctive from that amplified from the genomic DNA of the soil solution; that refers to the specificity of the bacterial attachment to the nematode cuticle. Not all soil bacterial isolates were attached to the nematode cuticle; however, the 16S rRNA fragments of some isolates were amplified correspondingly with a low and high density from the DNA of the soil solution and the attached bacteria (Figure 2).

### 3.3. The Specificity of the Bacterial Attachment by Rothia sp. to P. neglectus Population

**Figure 3:**
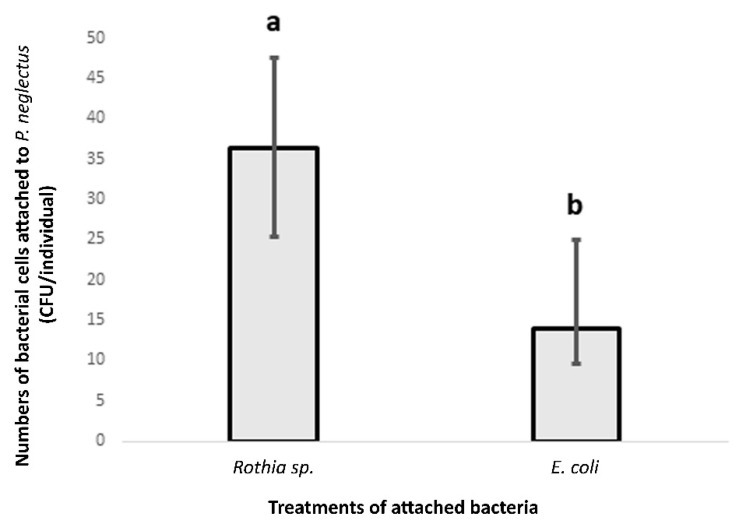
The bacterial attachment of *Rothia* sp. to the nematode cuticle of the *P. neglectus* population (Lachadella/France). *E. coli* was used as a control treatment. The nematodes were baited in the bacterial solution (OD_600_ = 0.12) overnight. Ten washed nematodes for each replicated sample were plated on the R2A medium and incubated at 28 °C. Counting the formed CFU was two days after incubation. Different letters indicate significant differences in the counts of CFU/individual between bacterial treatments (Wilcox test, *p* < 0.05, *n* = 8).

Compared to *E. coli*, considerable counts of alive bacterial cells from *Rothia* sp. were attached to the nematode cuticle of the *P. neglectus* population (Lachadella) (Figure 3).

The CFU counts resulting from plating the incubated nematodes in the R2A medium were higher in the treatment of *Rothia* sp. than that of *E. coli*, providing evidence of the specificity of the attachment by *Rothia* sp. to *P. neglectus*. 

In the *Rothia* treatment, the values of CFU/individual ranged between 14 and 86, referring to the variability in the attachment level of *Rothia* sp. among individuals within the same nematode population (Figure 3).

### 3.4. The Attachment by Rothia sp. to Four Populations of P. neglectus

**Figure 4:**
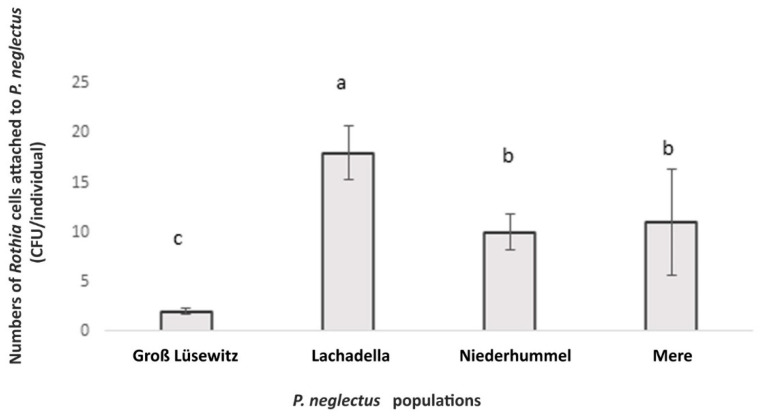
The bacterial attachment by *Rothia* sp. to the nematode cuticle of *P. neglectus* originated from four populations. Surface-sterilized nematodes were incubated overnight in the bacterial suspension (OD_600_ = 0.12). Evaluating the CFU happened two days after plating the nematodes on the R2A medium. Different letters indicate significant differences in the counts of CFU among populations (Dunn’s test, *p* < 0.05, *n* = 8).

In an assay to test the attachment by *Rothia* sp. to four geographically different populations of *P. neglectus* (Groß Lüsewitz, Lachadella, Niederhummel, Mere), the numbers of alive attached bacterial cells were intra- and inter-variable. The average number of CFU evaluated by plating the incubated nematodes on R2A was the highest in the Lachadella population and lowest in the Groß Lüsewitz population (Figure 4).

No correlation appeared between the attachment level of *Rothia* sp. and the geographic origin of the population. While they were similar between Niederhummel and Mere originating from Germany and the United Kingdom, the numbers of alive attached bacterial cells differed between Groß Lüsewitz and Niederhummel populations originating from Germany (Figure 4).

### 3.5. Penetration of P. neglectus Populations into Barley Roots with and without an Attachment by Rothia sp.

**Figure 5:**
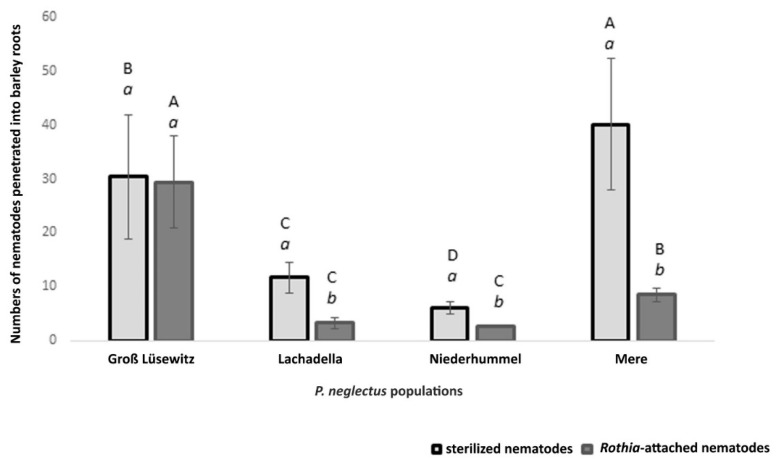
Numbers of nematode individuals from four populations of *P. neglectus* penetrated barley roots with or without an attachment by *Rothia* sp. 200 nematodes were added to a three day seedling placed into a petri dish containing Pluronic F127 gel and incubated at 22 °C and 60% relative humidity for seven days. The counting was for the nematodes that penetrated the roots stained by acid fuchsin. (a, b) compare the mean numbers within a population among treatments. (A, B) compare the mean numbers within the same treatment among the populations. Different letters indicate significant differences in the numbers of penetrated nematodes between treatments (a, b) or among populations (A, B) (Dunn’s test, *p* < 0.05, *n* = 8).

The bioassay conducted to test the nematode infectivity into barley roots showed that studied populations of *P. neglectus* are different in their aggressiveness. The average number of the penetrated sterilized nematodes (baited in sterilized water) was the highest in Mere and the lowest in the Niederhummel population (Figure 5).

Compared to the infection of sterilized nematodes, the bacterial attachment by *Rothia* sp. to the nematode cuticle caused reducing the penetration level of three from the studied populations (Figure 5).

The infection level of sterilized nematodes and *Rothia*-attached nematodes was higher in the Mere population than in Lachadella and Niederhummel. Even though the penetration levels of sterilized nematodes showed a significant difference between Lachadella and Niederhummel populations, the average numbers of infected *Rothia*-attached nematodes were similar between the two populations (Figure 5).

The bacterial attachment did not affect the root penetration by nematodes from the Groß Lüsewitz population (Figure 5).

### 3.6. Amplification of far-1 Gene of P. neglectus Populations for Sequence Determination

**Figure 6:**
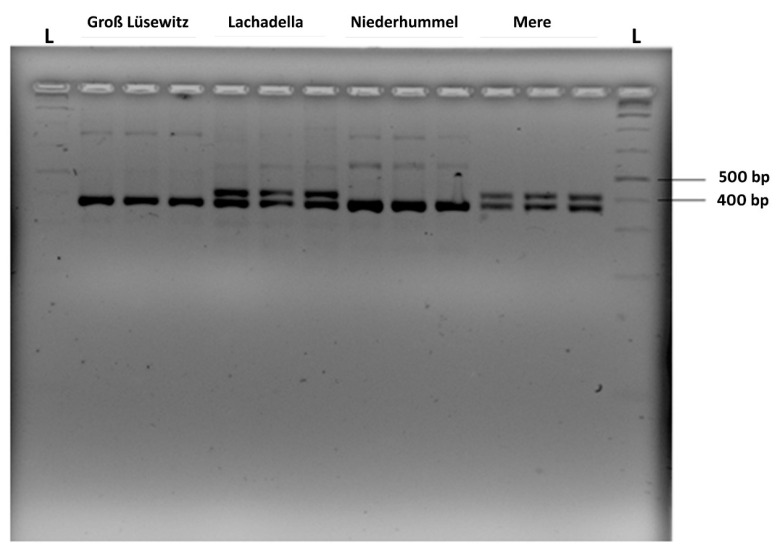
PCR products amplified by the primer pair Pn.far1f/Pn.far1r from the DNA of 200 pooled nematode individuals for each replicate from four populations of *P. neglectus*. The separation of PCR products was in 3% high-resolution agarose gel electrophoresis. L: 1 kb ladder.

The *far-1* gene fragments were amplified successfully from the pooled DNA of 200 nematodes per replicated sample for each of the four nematode populations (Figure 6).

While for Groß Lüsewitz und Niederhummel the PCR products appeared on a high-resolution agarose gel with one size, the amplified gene segments appeared in two sizes within each of the Lachadella and Mere populations (Figure 6). Sequencing of the cloned gene fragments revealed that the sizes of PCR-amplified *far-1* segments were 368 bp and 431 bp (Supplementary Figure S1).

The multiple sequence alignments of 18 cloned *far-1* segments revealed the variance in the gene sequence within and among populations of *P. neglectus* (Supplementary Figure S1). The gene similarity within a population can be less than amongst others. Four cloned fragments originated from Mere, Groß Lüsewitz and Niederhummelwere, identical in their sequences. The percentage similarity was 97–99% among 16 *far-1* fragments with 368 bp sequence length. Two gene variants originating from Lachadella and Mere had 431 bp sequence length and were only similar by 38–42% to the other gene segments.

**Figure 7:**
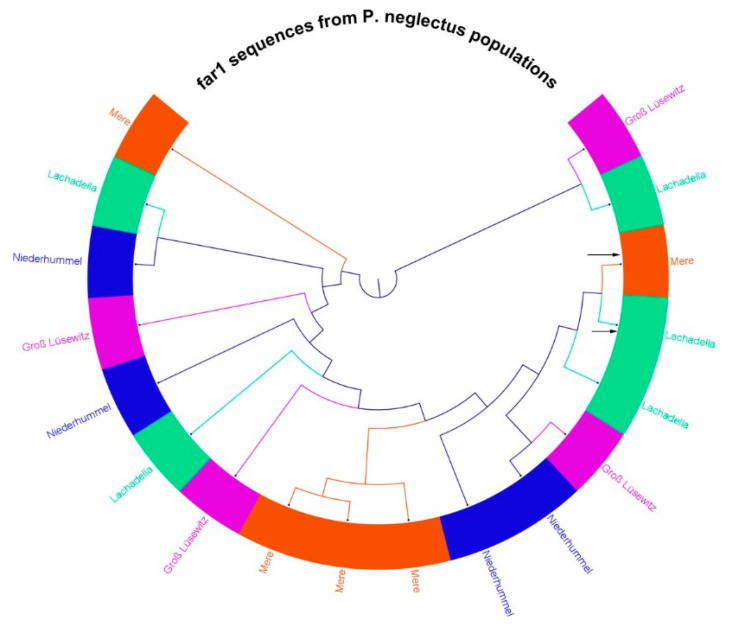
A cladogram of a phylogenetic tree of a multiple sequence alignment for 18 *far-1* segments derived from four geographic populations of *P. neglectus* (Neighbor-Joining tree with 1000 bootstraps generated in CLC 7.8). Two gene segments in Lachadella and Mere (referred to by arrows) had 431 bp sequence length. The other sixteen segments had 368 bp. Each color represents sequences from one population.

A phylogenetic tree showed that *far-1* sequences derived from geographically diverse populations were related more or less than those of the same (Figure 7). For example, *far-1* gene sequences from Gross Lüsewitz are related to that from Niederhummel more than to the gene sequences of the same population. However, the relatedness of *far-1* gene sequences within the Mere population was higher than that among Mere and others.

## 4. Discussion

The different geographical populations of *P. neglectus* differed in their bacterial attachment. As hypothesized, geographic nematode populations display discriminative cuticle and genetic structures, determining the specificity of nematode-microbes interaction. The nematode cuticle structure is highly variable, not only among different genera but also between the developmental stages within a species [2,30,31]. The cuticle components such as collagen proteins display high variability among nematode individuals of a particular species. For example, 122 collagen genes may be in the genome of *M. incognita* [32]. Collagen is considered one of the main cuticle components related to microbial association [33,34]. Carbohydrates on nematode surfaces might play a role in nematode-plant and nematode-microorganisms interactions [35,36]. The alteration in the nematode surface coat glycans that are considered surface antigens or receptors might help explain the host specificity observed in the attachment of *Pasteuria* endospores to the cuticles of root-knot nematodes [12,37,38,39]. The inconstancy in the cuticle compounds is mirrored in the nematode-microbes interaction since the nematode cuticle is the first active defense line towards hostplant and microorganisms [40].

The attachment test showed that the bacterial isolate of *Rothia* sp. associated specifically to the cuticle of *P. neglectus* and acted so differently among populations of this nematode. Despite the attachment mechanism that might differ among bacterial species, the bacterial attachment by *P. penetrans* also differed among *Meloidogyne* populations [10]. The study conducted on *M. javanica*, *M. incognita*, and *M. hapla* showed that attachment specificity occurred at a sub-species level with high differences in the number of attached endospores among different populations of the same nematode species.

Due to the bacterial diversity in the soil solution, the difference among populations in the numbers of CFU formed by soil bacteria was higher than that in *Rothia* attachment.

The penetration of *P. neglectus* in barley roots cultivated in the Pluronic F-127 gel was estimated. Since the nematodes can freely move in three dimensions in response to the root exudates, the soil-like medium Pluronic F-127 gel provides a reasonable investigation of plant-nematode interaction [41,42].

The number of nematode individuals inside barley roots was less when *Rothia* attached to three populations of *P. neglectus*. However, *Rothia* attachment did not cause a reduction in the number of penetrated nematodes from the Groß Lüsewitz population. The low level of *Rothia* attachment to individuals from the Groß Lüsewitz population might cause a low level of bacterial infections in this population.

The difference in the level of *Rothia* attachment between the three populations (Lachadella, Niederhummel, and Mere) was not compatible with the ability of *Rothia* to reduce the number of penetrated nematodes. The number of *Rothia* cells attached to nematodes from Lachadella was higher than from Niederhummel, but this difference in the attachment did not cause a difference in the nematode penetration between both populations. Additionally, the numbers of CFU of attached *Rothia* were similar between Niederhummel and Mere, but this similarity in the attachment did not inhibit the difference in the number of *Rothia*-attached nematodes penetrated barley roots between these populations. The distinguishing between the attachment and penetration processes of the bacterium can explain this result. Attaching the cuticle is the first step of microbial infection [43]. The second step is the penetration into the nematode cuticle by a germ tube [44]. The nematodes might then mount a defense against the spore penetration of the cuticle [45,46]. For example, *Caenorhabditis elegans* increased the gene expression of antimicrobial peptides in the epidermis against the infection process by the pathogenic fungus *Drechmeria coniospora* [46]). The ability of a microbe to kill the nematodes relies on the susceptibility of the individuals to that microbe. Despite the difference in the bacterial attachment that might appear between treated nematodes, the ratio of bacterial infection can differ between these individuals [45].

In addition to its demonstrated role in reducing the number of penetrated nematodes, *Rothia* sp. is considered one of the plant growth-promoting rhizobacteria (PGPR) that alleviated the adverse effects of pest infestation and resulted in an increase in the plant biomass yield of infested plants [47]. This dual effect of *Rothia* sp. render it an antagonistic candidate bacterium for sustainable control of PPN.

The bioassay results showed that studied populations of *P. neglectus* are different in their aggressiveness towards the susceptible barley (cv. Monroe). Griffin 1991 [21] and Al-Khafaji et al. 2019 [48] reported that *P. neglectus* populations differ in the pathogenicity toward the host plant. The main reason for the distinction in pathogenicity is the genetic variation [22,49].

Since the process of microbial attachment to nematodes is related to the physical and structural characteristics of nematode cuticles [15,16,40], analyzing the interfered genes will elucidate reasons for the variability in this process among nematode populations. As introduced, one of the gene effectors associated with the microbial attachment to the nematode cuticle is the fatty acid- and retinol-binding protein (FAR-1) [24]. FAR proteins also play a critical role in the development and infection processes of plant-parasitic nematodes [23,24]; they inhibit the defense reaction by obstructing the gene expression related to jasmonic acid pathways in host plant [50]. The silencing of Mj-FAR1 in tomato hairy roots resulted in decreased infection of *M. javanica* [51]. Analysis of this gene might therefore add information to interpret the difference in the microbial attachment among nematode populations and the differences in their aggressiveness toward the host plant.

The size and sequence of PCR-amplified *far-1* fragments were different within and among populations. The degree of relatedness between *far-1* sequences did not always depend on the origin of these sequences. Consistent with a previous study, the high similarity of effector gene structures appeared between geographically close and occasionally among distant populations [49]. The interplay between dispersal and genetic adaptation shapes the genetic variation in parasitism genes within and among nematode populations [49]. Since it is involved in the parasitism process of PPN [24], the *far-1* gene can display a high variability within and among populations. Maybe the variability in the *far-1* gene is one of the reasons caused differences in the bacterial attachment among *P. neglectus* populations.

In conclusion, the differential bacterial attachment within and among *P. neglectus* populations is evidence of the specificity in the nematode-microbes interaction, leading to a distinct nematode-plant interplay. Intra- and inter-genetic differences in the effector genes are one of the causes underlying the variable nematode-microbe interaction. All in all, the attachment of beneficial bacterial species to nematode cuticle of *P. neglectus* occurs in a population-specific manner, providing a deeper understanding of the interaction between the nematodes and their natural enemies, which in turn will help increase the effectiveness of the use of these antagonists to control the plant-parasitic nematodes.

## Supplementary Materials

The following supporting information can be downloaded at: https://www.mdpi.com/article/10.3390/microorganisms10081524/s1, Figure S1: Sequence alignment of *far-1* fragments amplified from nematode DNA of four populations of *P. neglectus* with published *far-1* cDNA GenBank accessions KY312539.1. Gene variants with numbers /1, 2, 6, 7, 17/ were amplified from nematode DNA of Lachadella/ France, and /3, 4, 5, 8, 18/ from Mere/ United Kingdom. The other gene variants with numbers /9, 12, 13, 16/ and /10, 11, 14, 15/ were derived from the two German populations (Groß Lüsewitz and Niederhummel consecutively). Sorting the alignment was by the sequence similarity. Among four variants (8–11), the sequence identity reached 100%, and among 12 variants (1–7, 12–16), the similarity was between 97% and 99%. The two variants (17, 18) were similar only by 50% in their sequences which displayed a high variability (58–62%) compared to the other sixteen variants.
