# Supplementary material for: The Difference in the Bacterial Attachment among Pratylenchus neglectus Populations and Its Effect on the Nematode Infection
Source: Microorganisms. 2022 Jul 27;10(8):1524. doi: 10.3390/microorganisms10081524 (PMC9414941; doi:10.3390/microorganisms10081524)
Supplement: Supplementary file 1 [file microorganisms-10-01524-s001.zip › microorganisms-1822434-supplementary.pdf]

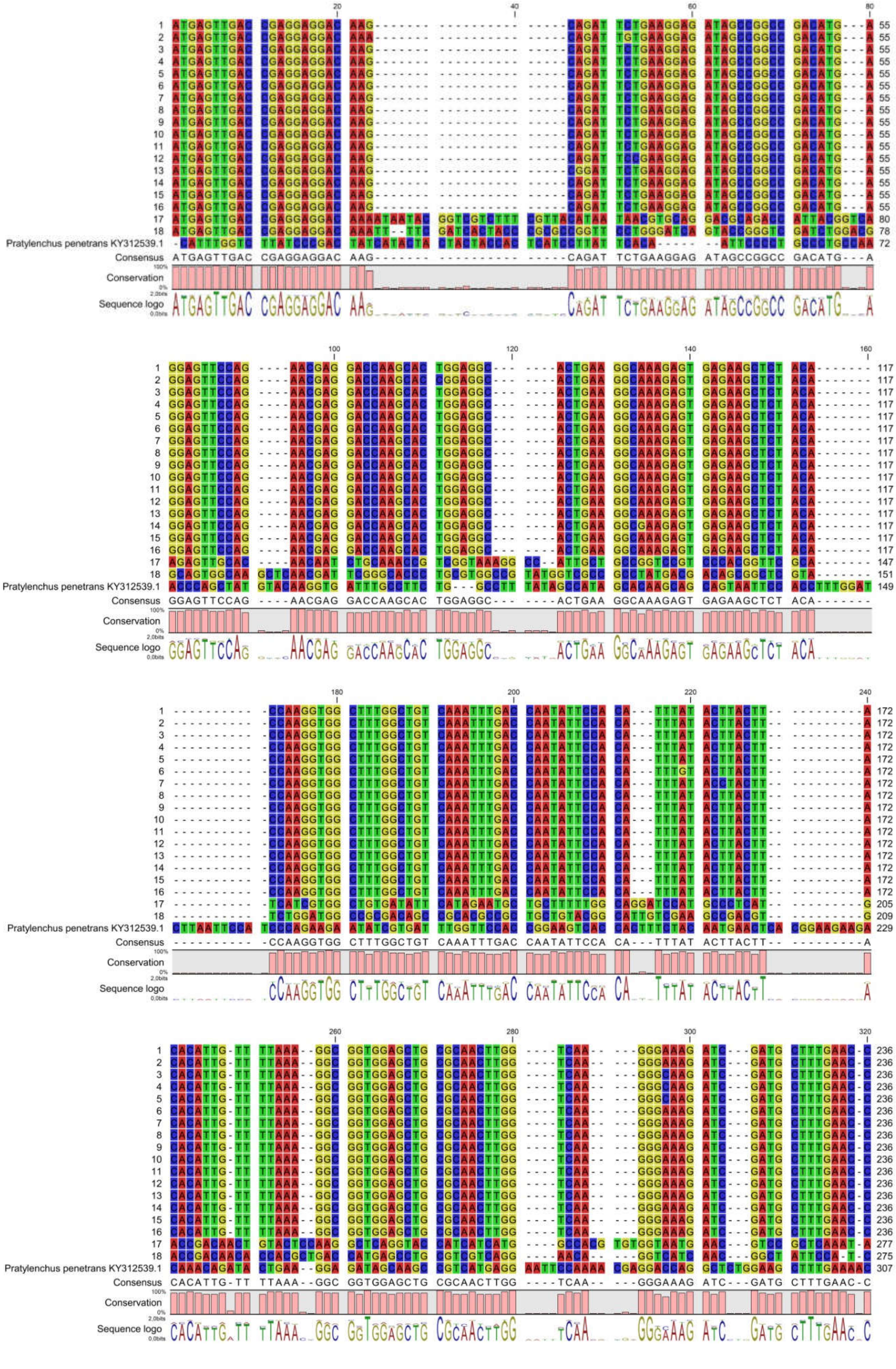
Pratylenchus penetrans KY312539.1
Consensus
Conservation
Sequence logo

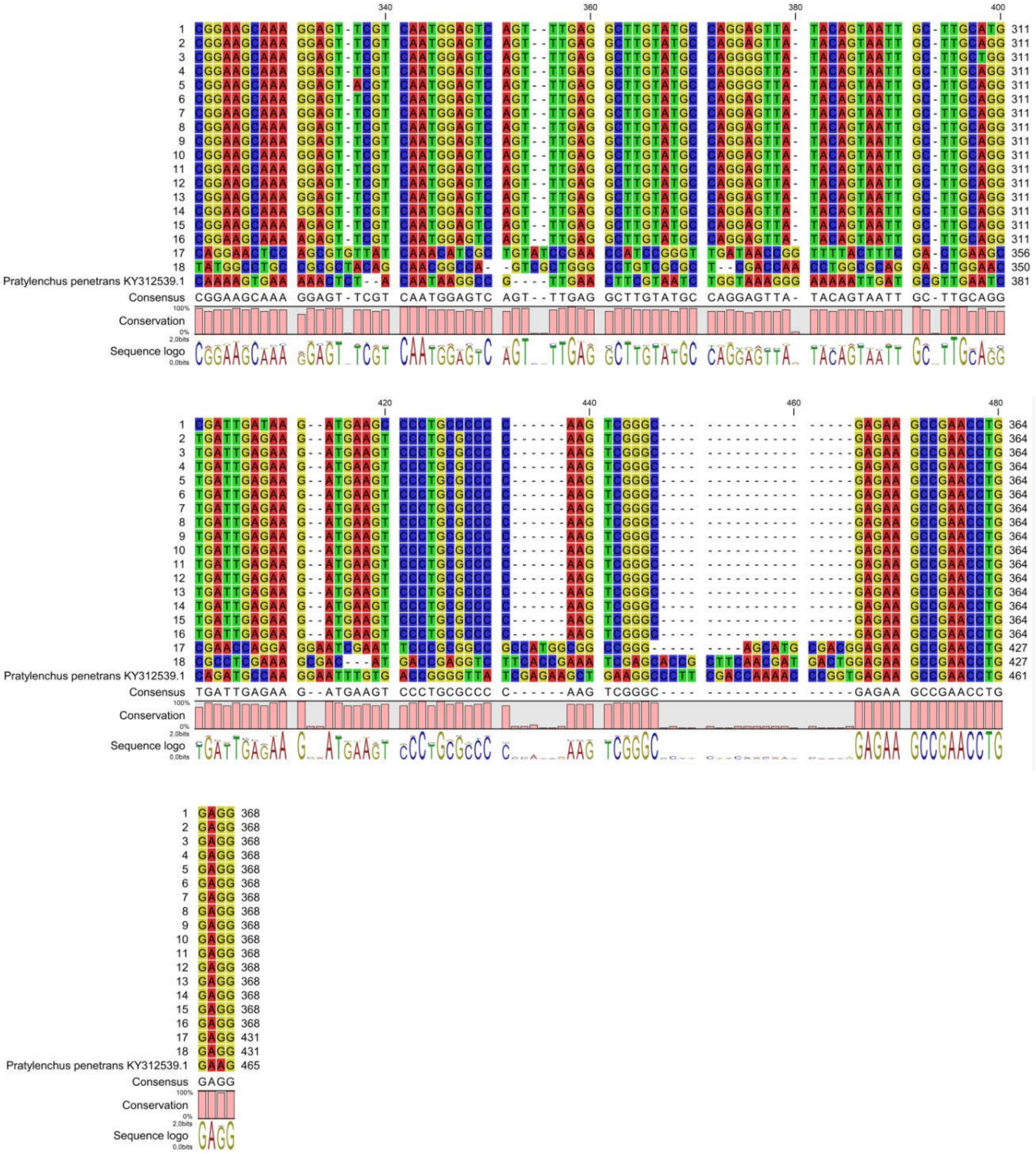

Figure S1. Sequence alignment of *far1* fragments amplified from nematode DNA of four populations of *P. neglectus* with published *far1* cDNA GenBank accessions KY312539.1. Gene variants with numbers /1, 2, 6, 7, 17/ were amplified from nematode DNA of Lachadella/ France ,and /3, 4, 5, 8, 18/ from Mere/ United Kingdom. The other gene variants with numbers /9, 12, 13, 16/ and /10, 11, 14, 15/ were derived from the two German populations (Groß Lüsewitz and Niederhummel consecutively). Sorting the alignment was by the sequence similarity. Among four variants (8-11), the sequence identity reached 100%, and among 12 varients (1-7, 12-16), the similarity was between 97% and 99%. The two variants (17, 18) were similar only by 50% in their sequences which displayed a high variability (58-62%) compared to the other sixteen variants.
